# A New Generation of Electrospun Fibers Containing Bioactive Glass Particles for Wound Healing

**DOI:** 10.3390/ma13245651

**Published:** 2020-12-11

**Authors:** Rachele Sergi, Valeria Cannillo, Aldo R. Boccaccini, Liliana Liverani

**Affiliations:** 1 Dipartimento di Ingegneria Enzo Ferrari, Università degli Studi di Modena e Reggio Emilia, Via P. Vivarelli 10, 41125 Modena, Italy; rachele.sergi@unimore.it (R.S.); valeria@unimore.it (V.C.); 2 Institute of Biomaterials, Department of Materials Science and Engineering, University of Erlangen-Nuremberg, Cauerstr. 6, 91058 Erlangen, Germany; aldo.boccaccini@fau.de

**Keywords:** chitosan, electrospinning, bioactive glasses, composite fibers, mechanical properties, wound healing

## Abstract

Chitosan fibers blended with polyethylene oxide (CHIT_PEO) and crosslinked with genipin were fabricated by electrospinning technique. Subsequently, CHIT_PEO bioactive glass composite electrospun mats were fabricated with the aim to achieve flexible structures with adequate mechanical properties and improved biological performance respect to CHIT_PEO fibers, for potential applications in wound healing. Three different compositions of bioactive glasses (BG) were selected and investigated: 45S5 BG, a Sr and Mg containing bioactive glass (BGMS10) and a Zn-containing bioactive glass (BGMS_2Zn). Particulate BGs (particles size < 20 μm) were separately added to the starting CHIT_PEO solution before electrospinning. The two recently developed bioactive glasses (BGMS10 and BGMS_2Zn) showed very promising biological properties in terms of bioactivity and cellular viability; thus, such compositions were added for the first time to CHIT_PEO solution to fabricate composite electrospun mats. The incorporation of bioactive glass particles and their distribution into CHIT_PEO fibers were assessed by SEM and FTIR analyses. Furthermore, CHIT_PEO composite electrospun mats showed improved mechanical properties in terms of Young’s Modulus compared to neat CHIT_PEO fibers; on the contrary, the values of tensile strain at break (%) were comparable. Biological performance in terms of cellular viability was investigated by means of WST-8 assay and CHIT_PEO composite electrospun mats showed cytocompatibility and the desired cellular viability.

## 1. Introduction

Electrospinning is a widely used technique to produce nano- and microfibers for various applications ranging from agriculture, food packaging to the biomedical field. This technique creates fiber mats with small fiber size, small pores, high porosity and large specific surface area [1]. Especially, the electrospinning technique is appropriate to obtain ultrafine and continuous fibers suitable for medical devices such as sutures, scaffolds [1], and chemical and biologically protective clothing, such as wound dressings. Furthermore, electrospun nanosized fibers imitate the native extracellular matrix (ECM), both its structure and features [2]. Moreover, the dimensions as well as the interconnectivity of the porosity provide suitable conditions for cells adhesion, growth and differentiation [3]. Therefore, electrospinning is an effective technique which permits to obtain nanofibers and microfibers suitable for tissue regenerative applications (i.e., hard and soft tissues). The spinnability and the morphology of electrospun fibers are influenced by different parameters such as parameters linked to polymer features (i.e., molecular weight, solubility), solution parameters (i.e., viscosity, electrical conductivity, surface tension) and other parameters (i.e., substrate properties, vapor and pressure of solvents and relative humidity) [2,4,5]. Among them, the polymer molecular weight, the concentration of the solution and the solvent exert the major influence on the features of the obtained fibers [6,7]. The solvent choice strongly contributes to determine the physical properties of the final polymeric solution. The dissolution of the polymer charged chain is favored by solvents with high dielectric constant which contribute to improve the conductivity of the solution and to reduce its voltage. Additionally, solvents with low boiling point evaporate faster from the surface of polymer allowing the formation of fibers when the voltage is applied. Acetic acid, formic acid or acetone are being increasingly considered as benign solvents, as reported previously [8], because they are less harmful than conventional solvents for electrospinning (i.e., chloroform, dichloromethane or methanol) [8,9]. Polysaccharides such as cellulose, alginate, hyaluronic acid, and chitosan are promising natural polymers to substitute synthetic polymers in various biomedical applications due to their renewable nature and their abundance [10]. Among natural polymers, chitosan has raised attention because of its unique structure which exhibits specific properties: biodegradability, non-toxicity, acceleration of tissue regeneration, hemostatic nature, cost-effectiveness and high availability [10]. Chitosan possesses excellent biocompatibility, which decreases with increasing (i) deacetylation degree (DD), (ii) solubility, and (iii) degradation rate [11]. The chemical structure of chitosan that contains hydroxy- and amino groups allows its chemical modification via an acid condensation reaction. For instance, condensation of chitosan with terpyridine-bearing molecules results in a variety of applications including the development of metal sensors [12,13]. Additionally, chitosan showed bactericidal activities attributed to its capacity to bind the sialic acid in phospholipids retaining the movements of microbiological substances [14]. These characteristics make chitosan promising for different biomedical applications such as artificial skin, tissue engineering, medical textiles, wound healing and drug delivery systems [15]. Unfortunately, chitosan exhibits low strength, high instability [16] and it is insoluble in water, alkali and most mineral acidic systems. However, chitosan is soluble in organic acid such as trifluoracetic acid, formic acid, acetic acid and lactic acid [17]. In this work, acetic acid was chosen and used for electrospinning, because it is considered a benign solvent [17].

Chitosan solubility in aqueous acidic media is attributed to amino groups; however, such groups make chitosan solution highly viscous, complicating its electrospinnability [18]. Moreover, the formation of hydrogen bonds once chitosan dissolves in acidic solution further challenges the electrospinning process [19]. This behavior limits the use of neat chitosan for electrospinning [20]. Therefore, polyvinyl alcohol (PVA) or polyethylene oxide (PEO) has been considered to be mixed with chitosan solution to improve the spinnability by increasing the chain entanglement and decreasing the electrical conductivity of the solution [21]. Blending chitosan with other polymers permits to obtain hybrid materials with better properties with respect to those of the individual polymers. In addition, chitosan electrospun fibers lose the fibrous structure once in contact with aqueous solutions. Thus, to preserve the integrity of the fiber structure, chitosan needs to be crosslinked [22] to avoid limitation in its use for biomedical applications [23]. In situ crosslinking [24] or post-crosslinking processes [25] are necessary to inhibit the solubility of electrospun chitosan fibers. Furthermore, crosslinking enhances tensile strength and elongation at break of fibers in wet conditions [26]. Crosslinkers stabilize polymers through the coupling and bonding of functional groups between polymer chains. The most commonly crosslinker for chitosan electrospun nanofibers is glutaraldehyde (GA), but its potential cytotoxicity represents the main concern for its use [22]. A valid alternative to the toxic GA is the use of genipin (GP), which is derived from the fruits of *Gardenia jasminoides Ellis* [22]. Genipin is stable, biocompatible and non-cytotoxic [22]. The crosslinking mechanism of chitosan is based on GP capacity to crosslink proteins and polysaccharides containing primary amine groups [27]. The crosslinking reaction varies at different pH values [28]; under acidic condition a nucleophilic attack by the amino group of chitosan on the olefinic carbon atom of genipin occurs [29].

In this work, chitosan/polyethylene oxide (CHIT_PEO) were mixed with acetic acid to fabricate fibers by electrospinning technique, adding genipin as crosslinker before electrospinning. The purpose was to use natural polymers to fabricate fibers suitable for tissue engineering applications and in particular wound healing because of their interactions with the host tissues [30]. Previous studies employed different polymers to blend chitosan to improve its spinnability as reported previously [31], and the combination of chitosan with other polymers has already been examined for wound dressings [32].

Additionally, since the electrospinning technique permits to incorporate various bioactive inorganic materials into polymer fibers [25,33], bioactive glass powders were added to enhance the bioactivity, biological and mechanical properties of fibers. Chitosan/PEO nanofibers containing bioactive glass micrometric particles (45S5 and SiO_2_:CaO:Na_2_O:P_2_O_5_) have been already fabricated by electrospinning, as reported in literature [34,35].

To the best of our knowledge, CHIT_PEO composite electrospun mats with Sr and Mg containing bioactive glasses and Zn-containing bioactive glasses have not been fabricated yet. To this purpose, we decided to incorporate in CHIT_PEO fibers two novel bioactive glasses: BGMS10 [36,37,38,39] and BGMS_2Zn [37,40], which resulted to be very promising in various applications in terms of biological properties, also in contact with human dental pulp stems cells and human mesenchymal stem cells [39,41]. Furthermore, preclinical studies in animal models using BGMS10 and BGMS_2Zn particulates are on-going. Thus, these bioactive glasses were added for the first time to CHIT_PEO fibers to fabricate CHIT_PEO composite electrospun mats with the expectation to improve tissue regeneration. 45S5 bioactive glass [42] was also used as a control to fabricate CHIT_PEO composite electrospun mats. Electrospinning parameters were optimized to obtain both CHIT_PEO fibers and CHIT_PEO composite electrospun mats; subsequently, microstructural characterization, mechanical testing, in vitro bioactivity, and biological tests were performed to investigate the efficiency of both CHIT_PEO fibers and CHIT_PEO composite electrospun mats.

## 2. Materials and Methods

### 2.1. Preparation of Glass Powder

Commercial raw powders (Carlo Erba, Milano, Italy) were separately mixed for 3 h in a jar and then melted in a platinum crucible in air. The bioactive glasses were melted at 1450 °C for 45 min, by a classic melt-quenching route as reported in [43,44,45]. The molten glass was quenched in water (at room-temperature) to obtain a frit, which was dried at 110 °C. Subsequently, the glass powders were obtained by milling the frit of each glass composition. The composition (in mol%) of each bioactive glass is listed in Table 1. Then, before the electrospinning process, the glass powders were incorporated into polymeric solution.

### 2.2. Solution Preparation

Chitosan (CH, medium molecular weight, Sigma Aldrich, Taufkirchen, Germany) 3% *w/v* in aqueous acetic acid (AA, VWR, Darmstadt, Germany) solution and polyethylene oxide (PEO, Mw 900000, Sigma Aldrich, Taufkirchen, Germany) 3% *w/v* in aqueous acetic acid (AA, VWR, Darmstadt, Germany) solution were mixed at ratio 90/10 and 95/5 and stirred for 48 h before the electrospinning process.

For the fabrication of CHIT_PEO_45S5, CHIT_PEO_BG10 and CHIT_PEO_BGZn composites, 20% wt of bioactive glasses (45S5, BGMS10 and BGMS_2Zn) with respect to the total polymeric amount was added and stirred 10 min before electrospinning.

### 2.3. Crosslinking of Electrospun Fibers

Genipin (≥98% (HPLC), powder, Sigma Aldrich, Taufkirchen, Germany) was used as crosslinker [46]. To obtain the crosslinking of chitosan fibers, 10% *w/v* genipin solution was prepared by dissolving genipin powders in ethanol (98%, VWR) and it was stored at +4 °C. The CH/genipin weight ratio in the solutions was 3%wt of genipin respect to chitosan amount; the volume of genipin solution was added at room temperature to CHIT_PEO acetic acid solution and stirred for 5 min before electrospinning [47]. For composite electrospun mats containing bioactive glass powders (45S5, BGMS10 and BGMS_2Zn), genipin was added and mixed for 5 min before adding the bioactive glass powders (20%wt respect to the total polymeric amount). Then, the electrospun fibers and composites mats were exposed to water vapor at 37 °C for 24 h immediately after electrospinning, according to the protocol reported in the literature [47].

### 2.4. Electrospinning Process

The same electrospinning parameters were used to fabricate both neat CHIT_PEO fibers and CHIT_PEO composite electrospun mats (CHIT_PEO_45S5, CHIT_PEO_BG10 and CHIT_PEO_BGZn). Table 2 summarizes the parameters used to fabricate fibers with the amount of genipin used to crosslink fibers and the amount of bioactive glasses.

Briefly, 20 kV as voltage, 10 cm as distance between the needle tip (diameter 21G) and the target, and a flow rate of 3 mL/h were used during the process. A commercially available setup (Starter Kit, Linari engineering srl, Pisa, Italy) was used for electrospinning at temperature (T) in the range 25–28 °C and relative humidity (RH%) in the range 23–35%.

### 2.5. Microstructural Characterization and Mechanical Testing

The morphology of samples was investigated using a SEM microscope (FE-SEM-EDS, Auriga Base, Zeiss, Jena, Germany) after sputtering samples’ surface with gold (Sputter Coater, Q150T, Quorum Technologies, Darmstadt, Germany). Subsequently, ImageJ (NIH, Bethesda, MD, USA) was employed to measure the diameter of 50 fibers and 50 joints of each sample to calculate the average of fibers and joints diameters [48].

To investigate samples before immersion in SBF (Simulated Body Fluid), FTIR spectroscopy was performed (40 spectral scans, resolution: 4 cm^−1^, wave number range 1800–500 cm^−1^) using a spectrometer (Shimadzu, IRAffinity-1S, Fourier Transform infrared spectrophotometer, Kyoto, Japan).

Additionally, SEM analysis (ESEM Quanta 2000, FEI Co., Eindhoven, The Netherland) was performed after soaking samples in Simulated Body Fluid solution (see Section 2.6) to observe the eventual hydroxycarbonate apatite layer formation on the fiber surfaces, which is considered a marker of bioactivity [49].

Furthermore, CHIT_PEO fibers and CHIT_PEO composite electrospun mats were fixed in a paper frame for investigating their mechanical properties at room temperature using uniaxial tensile test (5960 Dual Column Tabletop Testing System, Instron^®^, Darmstadt, Germany), equipped with a load cell of 100N and a cross-head speed of 5 mm/min.

### 2.6. In Vitro Bioactivity

The procedure developed by Kokubo et al. [49] was followed to prepare the SBF (Simulated Body Fluid) solution. The ion concentration of the inorganic SBF mimics that of human body plasma. CHIT_PEO fibers and CHIT_PEO_45S5, CHIT_PEO_BG10 and CHIT_PEO_BGZn mats (0.8 × 0.8 cm^2^) were fixed on round scaffold supports (designed and printed with 3D Printer Ultimaker, Utrecht, Netherlands) before immersion in SBF (4 mL). Before SEM analysis, the samples were rinsed and dried 1, 7 and 14 days after incubation at 37 °C.

### 2.7. WST-8 Assay

Bone murine stromal cells ST-2 cell line (Leibniz-Institut DSMZ—German Collection of Microorganisms and Cell Cultures GmbH, Braunschweig, Germany) were seeded and kept in contact with samples for 1 and 7 days before performing WST-8 assay (CCK-8, Sigma Aldrich, Taufkirchen, Germany). All samples were fixed on sample holders designed and printed with a 3D Printer (Ultimaker, Utrecht, Netherlands) to fit inside a 48-multiwell plate. Before the cell seeding, the samples were disinfected by exposure to UV light for 1 h. Drop seeding was performed by using an inoculum ratio of 2.0 × 10^5^ cells/mL with a drop of 50 µl per sample. 1 mL of RPMI medium was added to each well 15 min after incubation [50]. ST-2 cells were cultured (in incubation at 37 °C with 5% CO_2_) in RPMI 1640 medium (Thermo Fisher Scientific, Erlangen, Germany) with the addition of 10% fetal bovine serum (Lonza) and 1% penicillin/streptomycin (Lonza), before the seeding.

### 2.8. Statistical Analysis

ANOVA one-way analysis was performed to evaluate the results of cell viability. A *p*-value < 0.05 was considered statistically significant.

## 3. Results and Discussion

### 3.1. Microstructural Characterization and Mechanical Analysis

**Figure 1:**
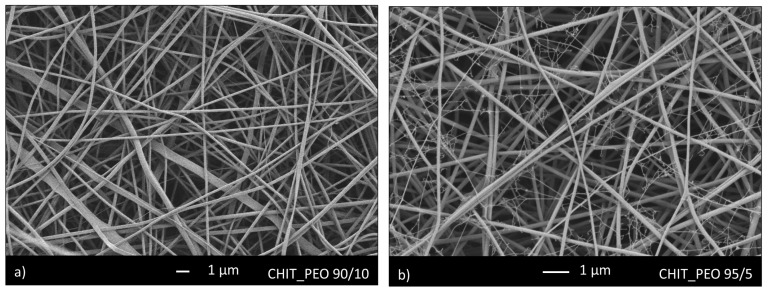
SEM analysis of CHIT_PEO fibers with ratio 90/10 (**a**) and 95/5 (**b**).

Initially, CHIT_PEO was produced using CHIT_PEO ratio of 90/10 and 95/5. SEM analyses of both CHIT_PEO 90/10 (Figure 1a) and CHIT_PEO 95/5 (Figure 1b) showed homogeneous fibers. CHIT_PEO with ratio 95/5 was chosen as starting polymeric solution to minimize the use of PEO as much as possible without compromising the spinnability of chitosan solution [51,52].

**Figure 2:**
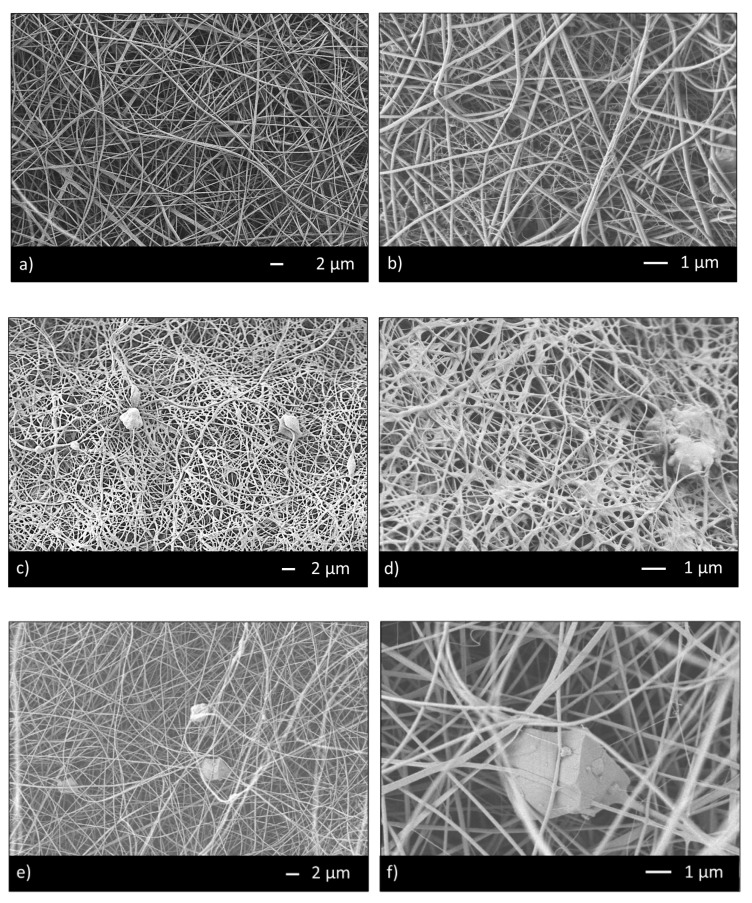

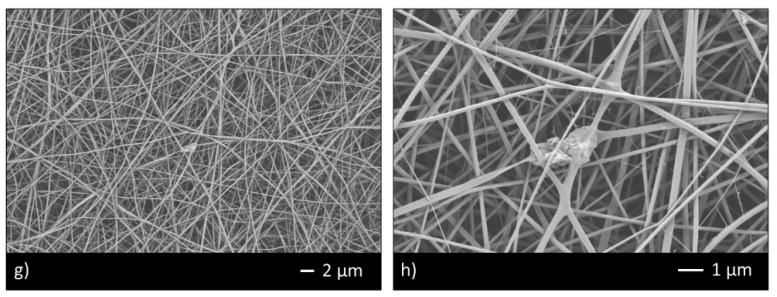
SEM analysis of CHIT_PEO (**a**,**b**); CHIT_PEO_45S5 (**c**,**d**); CHIT_PEO_BG10 (**e**,**f**) and CHIT_PEO_BGZn (**g**,**h**).

Thus, the ratio 95/5 was used to fabricate CHIT_PEO and CHIT_PEO composite electrospun mats (CHIT_PEO_45S5, CHIT_PEO_BG10 and CHIT_PEO_BGZn). CHIT_PEO, CHIT_PEO_45S5, CHIT_PEO_BG10 and CHIT_PEO_BGZn showed homogeneous fibers diameter distribution and CHIT_PEO composite electrospun mats showed some embedded bioactive glass particles (Figure 2a–h). Furthermore, joints between main fibers were visible in Figure 2b,f–h.

These interconnections between the main fibers and among the pre-formed joints are generated by electric poles which produce the main fibers. The electric poles do not only generate the main fibers but also the pre-formed joints creating other connection between these new joints (i.e., spider net) [53]. Furthermore, such joints could be attributed to a non-balancing effect between electrical forces and surface tension and to the environmental parameters (i.e., temperature and humidity during electrospinning) [54]. The average of fibers diameter and the joints diameter were calculated [48] (Table 3).

The results demonstrate that BGs particles did not influence the morphology of fibers in terms of both average fiber diameter and joints diameters. CHIT_PEO fibers and CHIT_PEO composites fibers are thinner compared to poly (ε-caprolactone) (PCL)/CH fibers previously obtained by using benign solvents [18,55]; the thinner diameter is probably ascribed to higher elongation forces induced by the charge density in the ejected jet. In fact, a reduced diameter of fibers was also detected by increasing the chitosan amount with respect to PCL as reported in [56].

**Figure 3:**
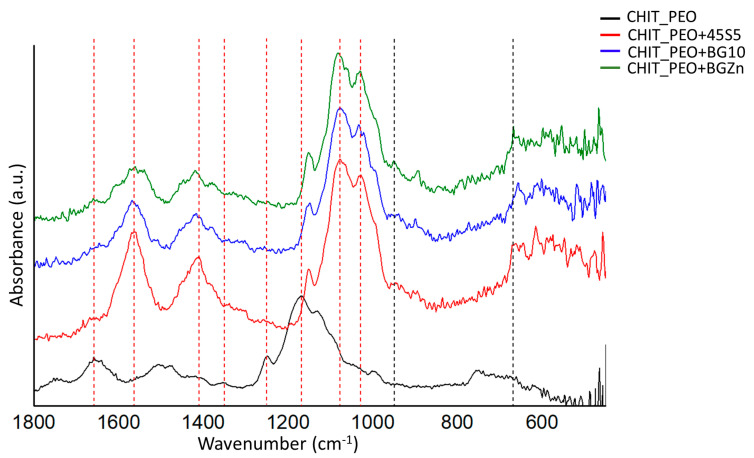
FTIR spectra of CHIT_PEO, CHIT_PEO_45S5, CHIT_PEO_BG10 and CHIT_PEO_BGZn electrospun mats before immersion in SBF solution. The characteristic bands of CH are marked by red dashed line, while bioactive glasses characteristic bands are marked by black dashed line.

FTIR analysis performed before soaking samples in SBF solution revealed the characteristic bands of chitosan and some corresponding to the bioactive glasses. The bands at 666 cm^−1^ could be ascribed to O-P-O bending [35] and at 949 cm^−1^ could be ascribed to Si-O stretching [34] of bioactive glasses (Figure 3).

The characteristic bands of chitosan are marked by red dash lines in Figure 3: the bands at 1658 and at 1560 cm^−1^ correspond to amide I and amide II, respectively [54]. The bands at 1416, 1348 and 1248 cm^−1^ were ascribed to CH_2_ bending, CH_2_ wagging and CH_2_ symmetric twisting, respectively [34]. The band at 1167 cm^−1^ can be ascribed to oxygen stretching band [54]. Finally, the bands at 1080 and 1028 cm^−1^ can be attributed to C-O stretching [34,54].

Mechanical properties of CHIT_PEO, CHIT_PEO_45S5, CHIT_PEO_BG10 and CHIT_PEO_BGZn were evaluated by uniaxial tensile test. The values of tensile at break (%) and Young’s Modulus are detailed in Table 4.

**Figure 4:**
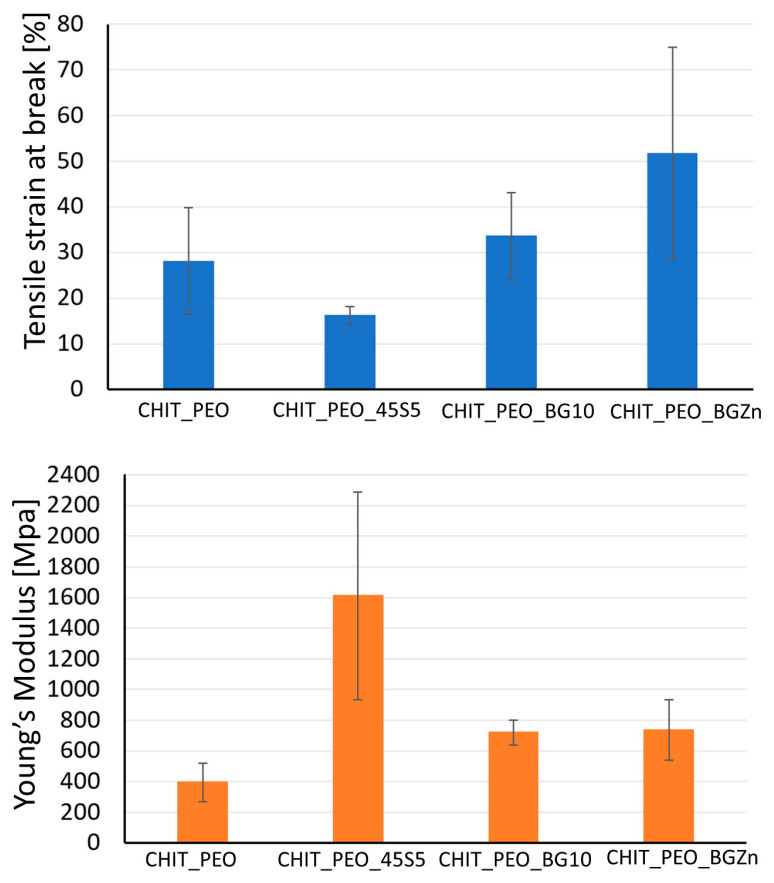
Tensile strain at break [%] and Young’s modulus [MPa] of CHIT_PEO, CHIT_PEO_45S5, CHIT_PEO_BGMS10 and CHIT_PEO_BGMS_2Zn.

Values of tensile strain at break (%) of CHIT_PEO composite electrospun mats are quite comparable to neat CHIT_PEO fibers, used as control. Although the Young’s Modulus of CHIT_PEO composite electrospun mats (Figure 4) is not significantly higher (*p* > 0.05) with respect to that of CHIT_PEO fibers, the findings suggest that the incorporation of bioactive glass particles could slightly improve the Young’s Modulus.

Generally, the incorporation of particles (i.e., bioactive glass) into polymers causes a decrease in the strain at break [57] because particles weaken the structure of the system by acting as rigid inclusions. Indeed, a decrease in the values of Young’s Modulus and tensile strain (%) of polycaprolactone/chitosan bioactive glass (PCL/CH_BG) composites compared to neat PCL/CH was observed [8]. The decrease could be due to the high amount of bioactive glass particles embedded (30% wt respect to the polymers amount) which was required to preserve the fibers bioactivity. On the contrary, in some cases an increase in the elongation at break was registered after the addition of particles [35,57,58] as for chitosan/PEO bioactive glass composites [35] and for chitosan/PEO silver particle composites [57,58]. This behavior could be ascribed to the formation of secondary bonds between BG particles and the matrix [35]. As reported in literature for other composite electrospun fibers [59], to further improve the mats mechanical properties, another possibility could be the incorporation of nanosized BG particles with the same composition.

### 3.2. In Vitro Bioactivity Investigations

**Figure 5:**
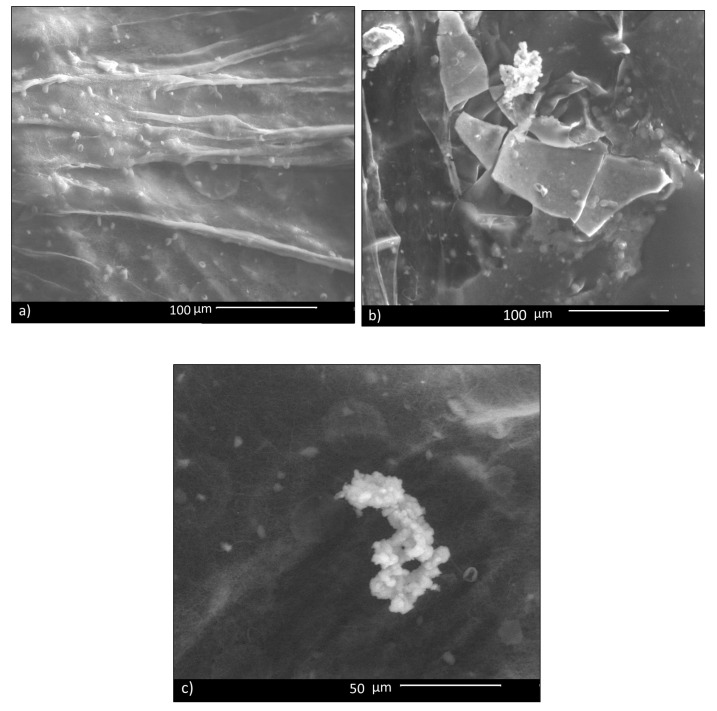
SBF test: 14 days after immersion of CHIT_PEO_BG10: in most parts, no HCA deposit is visible (**a**); in (**b**,**c**), a few isolated deposits are visible.

In vitro bioactivity of CHIT_PEO fibers and CHIT_PEO composite electrospun mats was investigated by immersion in SBF; this test assesses the capability of biomaterials to bond to bone by estimating the nucleation ability of hydroxycarbonate apatite (HCA) on samples’ surface. Such in vitro bioactivity test is generally performed for bone tissue applications and its role and relevance for soft tissues applications is still a matter of debate in the literature [60]. Some studies have shown that the HCA layer on biomaterials’ surface can lead to undesired calcification of soft tissues [61]; on the other hand, the HCA layer favors the formation of a strong bond between biomaterials and soft tissue, as reported in literature [62]. Therefore, since some previous studies which developed materials for healing applications tested the in vitro bioactivity (as [63]), CHIT_PEO composite electrospun mats were tested here for comparison purpose. Despite the fact that bioactive glass particles are embedded in the mats, CHIT_PEO composite electrospun mats showed a very limited in vitro bioactivity, in terms of HCA formation. In fact, 14 days after soaking in SBF, just a few isolated precipitates of HCA were detected on CHIT_PEO_BG10 mats (Figure 5). The same situation was observed also for the other composite mats.

### 3.3. Biological Investigations

**Figure 6:**
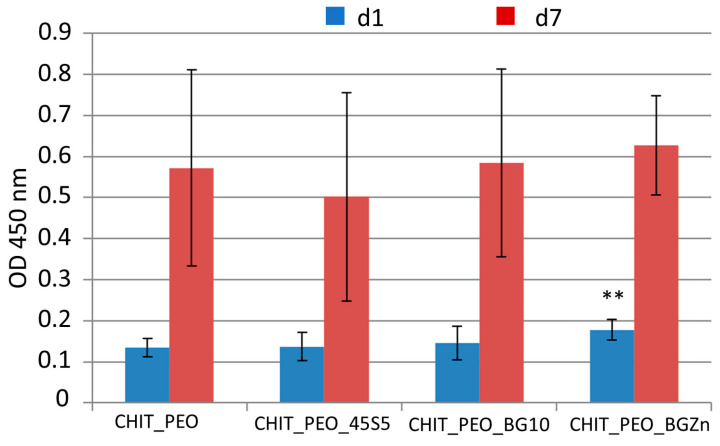
WST-8 assay: graph of OD at 450 nm for neat CHIT_PEO electrospun fibers and CHIT_PEO composite electrospun mats 1 and 7 days after seeding using bone murine stromal cells ST-2. ** *p* < 0.05 (ANOVA one way).

At 1 day, CHIT_PEO_BGZn has higher cellular viability respect to that of CHIT_PEO (*p* < 0.05) as shown by WST-8 assay (Figure 6). On the other hand, 7 days after seeding CHIT_PEO_BG10 and CHIT_PEO_BGZn showed slightly higher OD values compared to CHIT_PEO (but not statistically significant).

The presence of SrO, MgO and ZnO in the bioactive glass composition is hypothesized to enhance cellular viability, following reports in the literature [64]. In fact, Sr, Mg and Zn ions are considered therapeutic ions that can enhance tissue regeneration. In particular, Sr stimulates cell proliferation and angiogenesis [65,66]; Mg stimulates the migration and proliferation of microvascular cells [61]. Additionally, Zn stimulates wound healing [67] and angiogenesis [60]. Furthermore, Sr, Mg, and Zn ions are known to promote a specific cellular response, activating molecular signaling involved in the cell cycle [64,66]. Indeed, both BGMS10 and BGMS_2Zn resulted very promising compositions, suitable for the preparation of granules, scaffolds and composite systems, giving also very encouraging results in vitro in innovative 3D models with human mesenchymal stem cells [36,37,38,39,40,41].

Anyway, all the electrospun composite mats showed a good cellular viability and no potential cytotoxicity, and thus they could be safely used in contact with soft tissues for wound healing applications.

## 4. Conclusions

CHIT_PEO fibers crosslinked by genipin were fabricated by electrospinning technique after optimization of the processing parameters. Bioactive glass particles, namely 45S5, BGMS10 and BGMS_2Zn, were successfully incorporated into CHIT_PEO fibers, as shown by SEM and FTIR analyses. The incorporation of BG particles slightly improved the Young’s Modulus of the fiber mats, while the tensile strain at break (%) of CHIT_PEO composite electrospun mats was comparable to that of CHIT_PEO fibers.

Additionally, CHIT_PEO and CHIT_PEO composite mats showed limited bioactivity (i.e., just a few isolated precipitates of HCA were visible). However, as discussed, this result does not necessarily represent a drawback for composites developed for wound healing applications.

On the other hand, WST-8 assay showed that CHIT_PEO and CHIT_PEO composite mats are non-cytotoxic. In particular, 1 day after seeding, CHIT_PEO_2Zn showed higher OD value (p < 0.05) compared to CHIT_PEO fibers. Further studies incorporating different and higher concentration of BGs should be explored to identify the ad hoc concentration of these particular compositions of BGs to achieve enhanced biological performance.

Furthermore, in vivo animal tests should be performed in the future to corroborate the preliminary results obtained in this in vitro study.

## Figures and Tables

**Table 1:** Bioactive glass composition in (mol%).

Composition (mol%)			
Oxides	45S5 [42]	BGMS10 [36]	BGMS_2Zn [40]
**Na_2_O**	24.4	2.3	2.3
**K_2_O**	0	2.3	2.3
**CaO**	26.9	25.6	25.6
**MgO**	0	10	8
**SrO**	0	10	10
**ZnO**	0	0	2
**P_2_O_5_**	2.6	2.6	2.6
**SiO_2_**	46.1	47.2	47.2

**Table 2:** Electrospinning parameters.

Electrospinning Process Parameters	CHIT_PEO	CHIT_PEO_45S5, CHIT_PEO_BG10, CHIT_PEO_BGZn
**Solution Concentration (% *w/v*)**	3	3
**Genipin (%wt Respect to Polymeric Amount)**	3	3
**Bioactive Glass (%wt Respect to Polymeric Amount)**	0	20
**Solvent**	Aq. solution acetic acid (80%)	Aq. solution acetic acid (80%)
**kV**	20	20
**Distance Needle Tip-Collector (cm)**	10	10
**Needle Diameter (G)**	21	21
**Flow Rate (mL/h)**	3	3
**Temperature (°C)**	25–28	25–28
**Relative Humidity (%RH)**	23–25	23–25

**Table 3:** Summary of the average fibers’ diameter and the average of joints diameter.

	Average Fiber Diameter (nm)	Average Joint Diameter (nm)
**CHIT_PEO**	140 ± 40	47 ± 20
**CHIT_PEO_45S5**	170 ± 70	47 ± 20
**CHIT_PEO_BG10**	170 ± 60	42 ± 20
**CHIT_PEO_BGZn**	120 ± 40	41 ± 10

**Table 4:** Summary of the average of tensile strain at break and Young’s Modulus.

	Tensile Strain at Break [%]	Young’s Modulus [MPa]
**CHIT_PEO**	28 ± 12	396 ± 127
**CHIT_PEO_45S5**	16 ± 2	1611 ± 678
**CHIT_PEO_BG10**	34 ± 2	810 ± 81
**CHIT_PEO_BGZn**	52 ± 23	737 ± 522
